# Vision Affects Gait Speed but not Patterns of Muscle Activation During Inclined Walking—A Virtual Reality Study

**DOI:** 10.3389/fbioe.2021.632594

**Published:** 2021-04-09

**Authors:** Amit Benady, Sean Zadik, Oran Ben-Gal, Desiderio Cano Porras, Atalia Wenkert, Sharon Gilaie-Dotan, Meir Plotnik

**Affiliations:** ^1^Center of Advanced Technologies in Rehabilitation, Sheba Medical Center, Ramat Gan, Israel; ^2^St George’s University of London Medical School, Sheba Medical Center, Ramat Gan, Israel; ^3^School of Optometry and Vision Science, Faculty of Life Sciences, Bar-Ilan University, Ramat Gan, Israel; ^4^Brightlands Institute for Smart Society (BISS), Maastricht University, Maastricht, Netherlands; ^5^UCL Institute of Cognitive Neuroscience, London, United Kingdom; ^6^The Gonda Multidisciplinary Brain Research Center, Bar-Ilan University, Ramat Gan, Israel; ^7^Department of Physiology and Pharmacology, Sackler School of Medicine, Tel Aviv University, Tel Aviv, Israel; ^8^Sagol School of Neuroscience, Tel Aviv University, Tel Aviv, Israel

**Keywords:** locomotion, inclined surfaces, visuomotor integration, electromyography, visual dependency, rod-and-frame

## Abstract

While walking, our locomotion is affected by and adapts to the environment based on vision- and body-based (vestibular and proprioception) cues. When transitioning to downhill walking, we modulate gait by braking to avoid uncontrolled acceleration, and when transitioning to uphill walking, we exert effort to avoid deceleration. In this study, we aimed to measure the influence of visual inputs on this behavior and on muscle activation. Specifically, we aimed to explore whether the gait speed modulations triggered by mere visual cues after transitioning to virtually inclined surface walking are accompanied by changes in muscle activation patterns typical to those triggered by veridical (gravitational) surface inclination transitions. We used an immersive virtual reality system equipped with a self-paced treadmill and projected visual scenes that allowed us to modulate physical–visual inclination congruence parametrically. Gait speed and leg muscle electromyography were measured in 12 healthy young adults. In addition, the magnitude of subjective visual verticality misperception (SVV) was measured by the rod and frame test. During virtual (non-veridical) inclination transitions, vision modulated gait speed by (i) slowing down to counteract the excepted gravitational “boost” in virtual downhill inclinations and (ii) speeding up to counteract the expected gravity resistance in virtual uphill inclinations. These gait speed modulations were reflected in muscle activation intensity changes and associated with SVV misperception. However, temporal patterns of muscle activation were not affected by virtual (visual) inclination transitions. Our results delineate the contribution of vision to locomotion and may lead to enhanced rehabilitation strategies for neurological disorders affecting movement.

## Key Points

• While walking on inclined surfaces, visual cues modulate gait as behaviorally expressed by changes in walking speed.
• These gait speed modulations are associated with subjective visual verticality misperception.
• During leveled walking, the intensity of muscle activation reflects walking speed, rather than visually directed behavior (i.e., inclination illusion).

## Introduction

In order to maintain stability during bipedal locomotion, the musculoskeletal system continuously adapts to changing walking conditions *via* a complex motor process that relies on multisensory integration (Wall-Scheffler et al., 2011; Kimel-Naor et al., 2017; Cano Porras et al., 2020). Particularly, walking on inclined surfaces leads to significantly different gait cycle-related patterns and magnitudes of activation in lower limb muscles, as compared with leveled walking. This is evident by changes in patterns and magnitude of increased (e.g., rectus femoris and tibialis anterior) or decreased (e.g., gastrocnemius muscle) activation of specific muscles during downhill walking (Lay et al., 2007; Alexander and Schwameder, 2016; Pickle et al., 2016) or by increased muscle activation (e.g., gastrocnemius, hamstring, and rectus femoris) during uphill walking (Alexander and Schwameder, 2016; Pickle et al., 2016; Janshen et al., 2017). Inclined walking involves adjustment to gravitational forces. The *exertion effect* (uphill walking) and the *braking effect* (downhill walking) have been previously described and quantified (Cano Porras et al., 2020). Briefly, during uphill walking, the exertion effect counteracts the gravitational deceleration and allows the walker to maintain roughly stable gait speed, which is lower than natural speed during leveled walking (Sun et al., 1996; Sinitski et al., 2015; Kimel-Naor et al., 2017). During downhill walking, the braking effect prevents gravitational-driven uncontrolled speeding-up and allows the walker to descend in a steady gait speed, either faster or slower than walking on a leveled surface (Sun et al., 1996; McIntosh et al., 2006; Kimel-Naor et al., 2017; Cano Porras et al., 2020). It is suggested that locomotor modulation following gravitational changes while walking is mediated through an “internal model of gravity” (Merfeld et al., 1999; Campos et al., 2014; Lacquaniti et al., 2014; Balestrucci et al., 2017) that integrates multisensory available cues such as vestibular, proprioceptive (aka body-based cues), and visual cues. The exact role of vision in this process is yet unclear. To evaluate the relative contribution (“weight”) of vision to the behavioral outcome of the internal model of gravity during locomotion, there is a need to manipulate visual cues independently of body-based cues. In a recent study, we found that vision modulates gait speed, postural adjustment, and spatiotemporal gait parameters shortly after transition to a virtual inclination for approximately 20 s (Cano Porras et al., 2020). Yet, it remains unclear whether individuals which are more dependent on visual inputs than others would respond by greater modulations in gait speed following a virtual visual inclination. Furthermore, it is unclear whether patterns of muscle activation are also affected by mere virtual inclinations.

Visual field dependence in the context of locomotion is considered as the level of reliance on visual cues in comparison to body-based cues (Isableu et al., 1998; Willey and Jackson, 2014). A common method to assess visual field dependency is through the rod and frame test which is assumed to estimate the extent of subjective misperception of visual verticality (Lopez et al., 2006; Isableu et al., 2008; Bagust, 2013). Individual differences in visual field dependency have been reported (Kaleff et al., 2011), and it has been suggested to relate to balance in patients and populations with balance-related disorders (Lord and Webster, 1990; Bonan et al., 2006, 2007; Crevits et al., 2007). In the present study, we aimed to explore whether the gait speed modulations triggered by mere visual cues after transitioning to virtual-inclined surface walking are accompanied by changes in muscle activation patterns. Our main hypothesis was that the changes in muscle activation following virtually induced braking and exertion effects would be typical to those triggered by veridical (gravitational) surface inclination transitions. Furthermore, we investigated whether the level of subjective visual verticality misperception was associated with the change in gait speed following virtual (visual) surface inclination change. Our second hypothesis was that the magnitude of visual modulation on gait speed during virtual surface inclination changes varies across people and may be related to an individual’s subjective visual misperception of verticality.

## Materials and Methods

### Participants

Twelve young, healthy adults (mean age ± SD: 26.53 ± 3.09 years old, six males) participated in this study. Exclusion criteria were cognitive limitations, physical and visual restrictions, and any sensorimotor impairments that could potentially affect locomotion or the ability to adhere to instructions. The Institutional Review Board for Ethics in Human Studies at the Sheba Medical Center, Israel, approved the experimental protocol, and all participants signed a written informed consent prior to entering the study.

### Apparatus

#### Virtual Reality System

Experiments were conducted in a fully immersive virtual reality system (CAREN High End, Motek Medical, Netherlands) containing a moveable platform with six degrees of freedom (Kimel-Naor et al., 2017). A self-paced treadmill was embedded in the moveable platform, allowing participants to adjust the treadmill speed according to their preferred walking speed (Plotnik et al., 2015).

#### Physiological Measures Recording Device—Electromyography

Electrical activity was recorded at 1,024 Hz (eego^TM^ sports, ANT Neuro, Netherlands) and preprocessed using MATLAB-based software programmed in our lab. Electromyography (EMG) signals were filtered (finite impulse response band-pass of 20–400 Hz) and full-wave rectified. We measured four right lower limb muscles: tibialis anterior right (TAR), gastrocnemius right (GCR), rectus femoris right (RFR), and hamstring lateralis right (HLR), as these muscles have been previously described to alter their activation pattern during uphill and/or downhill walking. Using surface EMG to measure the rectus femoris (RF) muscle most likely involves “picking up” activity from the adjacent vastus lateralis (VL) muscle, in what has been termed as “cross talk” (Nene et al., 2004; Barr et al., 2010). Therefore, we refer to the placement of the electrodes over the RF as representing the combined activity of both muscles which contribute together to knee extension. The location of electrodes followed the SENIAM guidelines. Initially, the precise area was shaved and cleaned by alcohol to remove dead skin and reduce the impedance. Then, wired pairs of self-adhesive bipolar electrodes were attached over the muscle bellies with 2 cm between electrodes. Prior to recording, a calibration stage was conducted by asking participants to precisely move the relevant muscle for confirming reliable activation patterns on the control monitor.

#### Virtual Reality Version of the Rod and Frame Test

The rod and frame test estimates subjective visual verticality misperception. Specifically, the test measures how visual perception of the orientation of a central bar (rod) is influenced by the orientation of a peripheral visual reference frame around it. Implementation of the test was conducted in our lab using Unity software and C# scripting. The participants sat upright wearing a virtual reality (VR) head-mounted display (HMD) (HTC VIVE, HTC; New Taipei City, Taiwan) and were told not to move or tilt their head during the test. The VR environment consisted of a white frame (occupying ∼16°× 16° of the visual field) rotated at a trial-specific orientation and a white rod (11° long) inside the frame, its center coinciding with the frame’s center, but with its own independent orientation. Both were presented on a black background (screen resolution was 1,920 × 1,080). A sequence of 28 trials was presented during which the frame was initially at one of seven possible random positions: 0/±10/±20/±30° (0 was vertical, ++ was clockwise). Each of these initial frame positions was presented four times (Bagust, 2005). In addition, the initial angle of the rod was randomized (sampled from 0 to 180° range distribution). The participants’ task was to orient the rod perpendicular (i.e., vertical) to the true horizon, regardless of the surrounding frame’s orientation. This was achieved by rotating the rod around its center in a clockwise or counterclockwise direction using the VR system’s remote control. Importantly, the surrounding frame was unchanged by this manipulation. Once the participants perceived it as being vertical, they responded by pressing a button on the remote control, which led to the clearing of the display and the beginning of another trial.

### Procedure

#### VR Rod and Frame Test

After filling the informed consent, the first part of the experiment was to perform the rod and frame test. First, the team began by assuring that the participant felt comfortable with the HMD. Next, the participant underwent a short practice trial to confirm that he/she fully understood the task. Following the practice trial, 28 test trials were conducted and measured accurately. It is critical to note that the test was not limited in time and typically lasted 10 min, including the practice trial.

#### Gait Trials in a Large-Scale VR System

##### Habituation period to walking in self-paced mode during leveled and inclined surfaces

**FIGURE 1:**
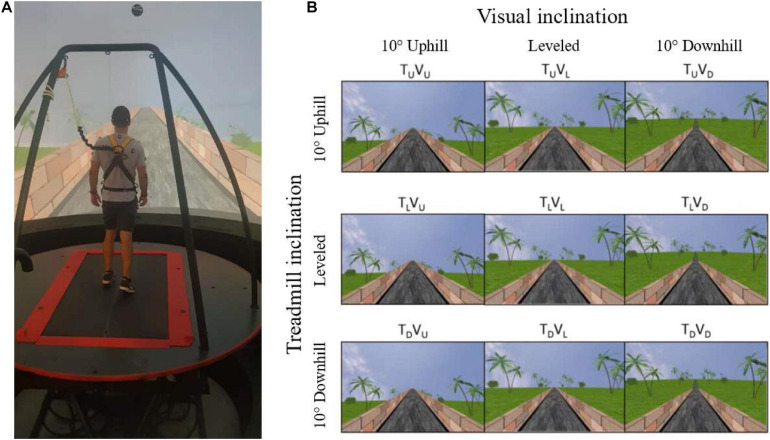
Apparatus **(A)** and the main experimental conditions **(B)**. **(A)** A fully immersive virtual reality system containing an embedded treadmill synchronized with projected visual scenes, wherein in this example, the treadmill is leveled, and the vision is uphill [see T_*L*_V_*U*_ in **(B)**]. **(B)** Main experimental conditions. Following leveled walking and after reaching steady-state velocity (SSV) and maintaining it for 12 s, a transition (5 s) occurred to one of the 11 different conditions (see text for details) presented in random order, lasting 65 s, in which the inclination of the treadmill (T) and/or visual scenes (V) transitioned to 10° uphill (_*U*_), remained leveled (_*L*_) or transitioned to -10° downhill (_*D*_). Rows represent treadmill inclination changes, and columns represent visual scene inclination changes. Visual scene inclination effect was achieved by the road appearing above (uphill), below (downhill), or converging (leveled) with the line of the horizon. In addition, the peripheral greenery is exposed more (downhill) or less (uphill) by the road. Vision–treadmill congruent conditions appear on the diagonal (T_*L*_V_*L*_ for continued leveled walking and T_*U*_V_*U*_ and T_*D*_V_*D*_ for uphill and downhill walking, respectively). Vision–treadmill incongruent conditions below the diagonal represent conditions with visual scene inclination more positive than the treadmill’s (T_*L*_V_*U*_, T_*D*_V_*U*_, T_*D*_V_*L*_), and above the diagonal visual scene inclination more negative than the treadmill’s (T_*U*_V_*L*_, T_*U*_V_*D*_, T_*L*_V_*D*_). See section “Materials and Methods” for more details.

A safety harness secured the participant to a metal frame on the moveable platform (Figure 1). The first part of the habituation was to familiarize the participant with the self-paced mode of the treadmill, which involved 10–15 min of leveled walking, aiming to provide practice of increasing and decreasing speed until he/she mastered the walking. The second part of the habituation involved connecting the EMG electrodes and calibrating the motion capture system. The third and final part of the habituation included one walking trial of each of the three possible inclinations (i.e., leveled, uphill, and downhill walking) when the visual and the physical cues were synchronized (“congruent” conditions; see more details below). Each trial lasted 3 to 4 min.

##### Gait experiments

The participants were informed that they would perform several short gait trials with short intervals between them. They were instructed to walk “as naturally as possible” and that “inclinations may occur during walking.” It is important to note that the condition did not affect the beginning stage of the trials, as every participant was explicitly instructed to begin from a standstill position and then progress into walking with both the treadmill and the visual scene leveled until reaching steady-state velocity (SSV, for definition, see *steady-state velocity* section below). Once reaching SSV, a 5-s-long transition of the treadmill and/or visual scene occurred (except in the congruent level condition, where no transition occurred). After transition, participants walked for an additional 65 s until the treadmill slowed down and stopped altogether. By convention, we refer to the transition start time as time zero (*t* = 0).

##### Experimental conditions

The protocol included 11 experimental conditions that the participant encountered in random order. Inclination of the treadmill (T) and/or visual scenes (V) transitioned to 10° uphill (U), remained leveled at 0° (L) or transitioned to −10° downhill (D). Figure 1 depicts the main 3 × 3 experimental design conditions where rows represent treadmill (T) inclination and columns represent visual scene (V) inclination (note that inclination of 10° is considered steep) (Proffitt et al., 1995). Treadmill and visual scene congruent conditions that served as baseline appear on the diagonal for leveled (T_*L*_V_*L*_), uphill (T_*U*_V_*U*_), and downhill (T_*D*_V_*D*_) walking. Visual scene–treadmill incongruent conditions include the following visual scene manipulations: for treadmill uphill inclination, the vision was leveled (T_*U*_V_*L*_) or downhill (T_*U*_V_*D*_); for treadmill leveled inclination, the vision was uphill (T_*L*_V_*U*_) or downhill (T_*L*_V_*D*_); and lastly, for treadmill downhill inclination, the vision was leveled (T_*D*_V_*L*_) or uphill (T_*D*_V_*U*_).

Since we expected to find the exertion and braking effects during virtual (visual) inclination transitions, which could possibly affect muscle activation patterns, we added two additional control conditions without treadmill or visual scene transitions. Participants were asked to increase (i.e., T_*L*_V_*L*_ increase) or decrease (i.e., T_*L*_V_*L*_ decrease) their speed voluntarily until the experimenter (AB) instructed them to maintain their current speed until the end of the trial. The target speeds were an increase of ∼15% and a decrease of ∼20% from individuals’ SSV, and the experimenter monitored this *via* the control computer. The quantitative values of the changing speeds were chosen to match the gait speed increase (in T_*L*_V_*U*_ condition) and the gait speed decrease (in T_*L*_V_*D*_ condition) observed in our previous study (Cano Porras et al., 2020). The purpose of these two conditions was to control the possibility that muscle activity pattern changes under virtual inclinations would be due to gait speed changes rather than to the change of gravitational forces.

### Outcome Measures

#### Gait Speed-Related Variables

To assess the post-transition effects on gait speed, we followed the methodologies introduced by Cano Porras et al. (2020). We looked at (i) the magnitude of the peak/trough of gait speed relative to the SSV (presented in %) and (ii) the time of this peak from the start of transition (seconds).

##### Steady-state velocity

A real-time algorithm monitoring treadmill speed determined SSV. According to the algorithm, SSV is attained after (1) a minimum 30 s of walking and (2) a consecutive period of 12 s with gait speed coefficient of variance less than 2%. Upon satisfying both conditions, the transition of the treadmill and/or visual scene inclination (as appropriate for the experimental condition) was automatically triggered.

##### Normalization of gait speed (see Supplementary Figure 1)

Normalization of gait speed (WS) in each experimental condition consisted of three steps. First, WS was divided by the averaged SSV (i.e., from the 12 s that defined the SSV period). In the new trace, the mean value of the 12 s SSV period is 1. The ratio between WS and the SSV was presented as a percentage. Finally, in order to clearly distinguish between the responses of increased and decreased velocity, the normalized trace was shifted so that the mean value of WS of the SSV period would be zero.

#### Individual Response Index to Virtual Inclines

To compute this metric, we used data from the incongruent T_*L*_V_*U*_, T_*L*_V_*D*_, T_*D*_V_*U*_, and T_*U*_V_*D*_ conditions. In each condition, the maximal speed change from the SSV was measured (i.e., the peak/trough, in %). We then calculated the average of the absolute values, and this was defined as the individual response index to virtual inclines for each participant.

#### Subjective Verticality Misperception Index

For each trial, the degree of deviation of the rod from the true vertical was measured and recorded as the position error. For each participant, the mean position error for seven different frame angles were calculated. Data from all participants were grouped by the frame angle (Bagust, 2005). We defined the rod and frame index as the average angle of deviation of the rod from the true vertical when the frame was projected at ± 20° (eight trials in total: four trials of +20° and four trials of −20°). This parameter allowed us to evaluate individual differences in gravitational misperception.

#### Electromyography Analysis

To recognize the effect of physical and virtual transitions on muscle activation patterns, we studied the patterns typical to the gait cycle, i.e., the interval between two consecutive heel strikes of the same leg. Therefore, gait cycle timing was first identified from the ground reaction force signals obtained by the force plates embedded beneath the treadmill’s belts. We identified the gait cycles 10 s before the transition (when the participant walked in SSV), during the 5 s of transition and 18 s post-transition (see Supplementary Figure 2). We examined 18 s post-transition as this is the time frame of gait speed modulations following the transition. We then chose by visual inspection five to nine cycles for the pre- and post-transition. The criterion of choice was to avoid residual effects of the 5 s of transition. It is critical to note that after the transition, the EMG pattern is constant throughout the selected gait cycles. We compared pre- to post-transition EMG patterns for each subject. For this, all selected gait cycle times were rescaled (0–100% of the gait cycle). From the time-scaled EMG traces, we grand averaged all the pre-data and post-data separately. With the averaged EMG traces, we computed the following two parameters:

(1) Magnitude—the summation (i.e., area under the curve) of the EMG traces.
(2) Pattern similarity of EMG activation pattern—For each participant and condition, the post-transition EMG trace was correlated with the pre-transition EMG trace (Pearson correlation). A high correlation value indicates a small change in the pattern of activation.

### Statistical Analyses

In order to test the differences between the walking conditions, we analyzed the muscle magnitude and EMG pattern separately. We verified that the data did not violate the normality assumption by running a Shapiro–Wilk normality test on all the EMG measurements of the magnitude of activation. We ran this test independently on each group that we examined, with a total of 56 normality tests. Among all groups, none indicated non-normal distributions (Shapiro–Wilk statistic 0.969 ≥ *p* ≥ 0.084). Then to analyze the magnitude of activation, we compared the mean of the magnitude summation of EMG activation (see above) for each muscle pre- and post-transition using a two-tailed paired *t*-test [significance was determined by dividing the *p*-value by four, to account for the four muscle comparisons; hence, *p*(corrected) = 0.0125]. To analyze the similarity of patterns, we initially calculated the correlation coefficients of each muscle between pre- and post-transition for each participant. Next, we applied the Fisher’s *Z* transformation to these correlation coefficients and ran a two-way repeated measures ANOVA with visual scene inclination and treadmill inclination as main factors. Pearson correlation coefficient was computed to evaluate the relationship between subjective verticality misperception index and the individual response index to virtual inclines. In order to compare between the magnitude of relative speed change following the transition, we conducted a one-way nonparametric Kruskal–Wallis test. Data were extracted using MATLAB R2017a (The MathWorks, Inc., Natick, MA, United States) and analyzed using SPSS software (v25, IBM).

## Results

### The Effects of Visual Cues on Walking in Physical and Virtual Inclinations

**FIGURE 2:**
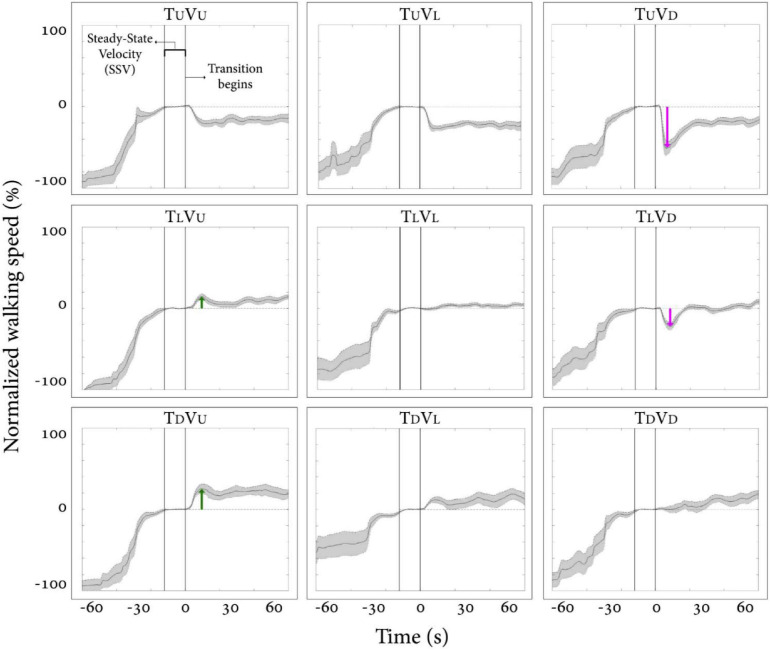
Post-transition adaptation of gait speed. Average self-paced gait speed (*N* = 12) relative to steady-state velocity (in %) for each condition. Time zero demarcates the end of the steady-state velocity period, after which a 5-s transition of the treadmill and/or visual scene occurred. Gray shading represents the standard error. Green arrows represent the peak of exertion effects in visual uphill walking, and red arrows represent the peak of braking effects in visual downhill walking. Irrespective of treadmill inclination upon transition, there is a tendency for a virtual (visual) downward transition to be followed by a decrease in gait speed and, to a lesser extent, for an upward visual transition to be followed by an increase in gait speed. Conditions: treadmill (T) and/or visual scenes (V) inclination transitioned to 10° uphill (U), remained leveled at 0°(L), or transitioned to −10° downhill (D).

We first examined how vision behaviorally influences gait speed for a short period after a visual surface inclination transition occurs, regardless of the veridical inclination (see Supplementary Video 1). Figure 2 depicts the average normalized self-paced gait speed relative to steady-state velocity for each condition, 1-min pre- and 1-min post-inclination transition. Briefly, in the congruent transition conditions (T_*U*_V_*U*_ and T_*D*_V_*D*_), there was a gradual monotonic change for downhill and a steep monotonic change for uphill walking. In contrast, the incongruent conditions were characterized by peaks (or troughs) in gait speed between 5 and 20 s after the virtual transition, which was determined by the vision–treadmill discrepancy direction (troughs when virtual inclination was lower than treadmill’s inclination, positive peak when virtual inclination was higher than treadmill’s inclination). Note that the vision–treadmill discrepancy direction was proportional to the vision–treadmill discrepancy magnitude, and this explains why a 20° discrepancy leads to a bigger peak than a 10° discrepancy.

The results in Figure 2 show how vision affects gait speed by dissociating vision from treadmill inclination. When treadmill inclinations were the same, we parametrically manipulated vision inclination and measured gait speed (see rows in Figure 2, where each row represents a fixed treadmill inclination change). This analysis indicates that visual input in the form of virtual inclinations affected and modulated gait speed in a parametric fashion 5–20 s after the virtual transition occurred when the treadmill transition was kept constant. These results are in line with our previous study (Cano Porras et al., 2020). For a full account on our specific results and comparison between the two studies, see Supplementary Material.

### Relation Between Visual Modulation of Gait Speed During Visual–Physical Incongruent Conditions and Subjective Misperception of Verticality

**FIGURE 3:**
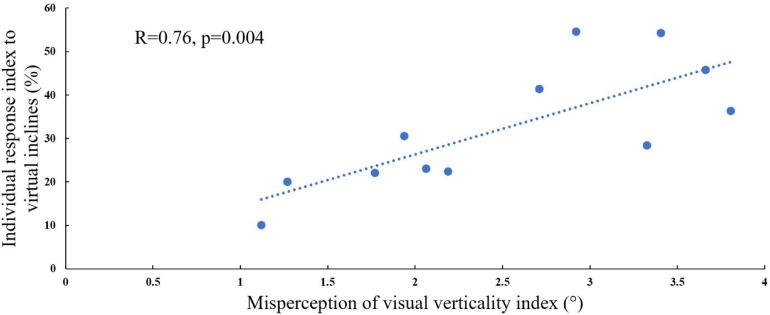
Visual field dependence relative to misperception of visual verticality. The extent of visual field dependence during locomotion with inclined surfaces is linked to the misperception of visual verticality. The *X*-axis represents the misperception of the visual verticality index assessed by the psychophysical rod and frame test while seated. The *Y*-axis represents the individual response index to virtual inclines based on the incongruent conditions, and each circle represents a participant (see section “Materials and Methods”). A significant correlation was found between these measures [Pearson’s *R* = 0.76, *p* = 0.004, *t*(10) = 4.075], suggesting that they may be relying on joint mechanisms. The dotted line represents the linear regression line *Y* = 0.472*X* + 0.1.

In order to address our second hypothesis that the magnitude of visual modulation on gait speed during virtual surface inclination changes varies across people and may be related to an individual’s subjective visual misperception of verticality, we calculated for each participant the magnitude of visual modulation on gait speed based on the gait speed changes in the incongruent conditions (i.e., individual response index to virtual inclines, see section “Materials and Methods” for calculation). An additional factor for our calculation was the index of subjective visual misperception of verticality estimated by the rod and frame illusion magnitude (see section “Materials and Methods”). We found a significant correlation when comparing these two independently obtained measurements [Figure 3; Pearson’s *R* = 0.76, *p* = 0.004, *t*(10) = 3.69], suggesting that they may rely on associated mechanisms.

### The Effects of Visual and Physical Transitions on Muscle Activation Patterns

**FIGURE 4:**
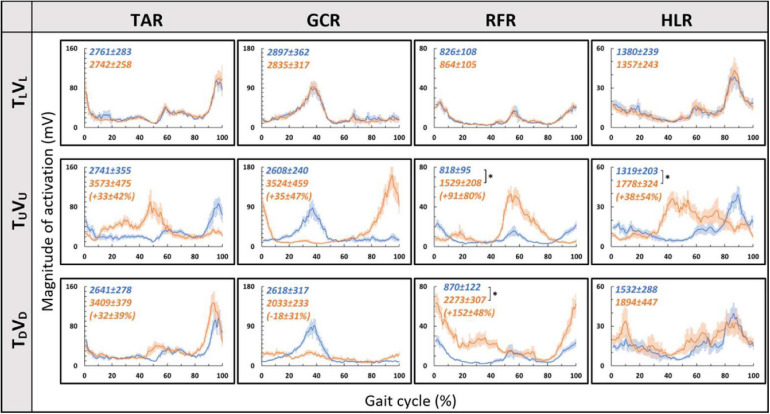
Pre- vs. post-EMG muscle activation patterns for visual–physical congruent transitions. Grand averages (across participants, *N* = 12) of muscle activation patterns (*Y*-axis, in mV) plotted against a time-normalized gait cycle on the *X*-axis (in % of the gait cycle) in blue (shaded areas represent SE) for pre-transition and in orange (shaded areas represent SE) for 20 s post-transition. Numerical values at the top left of each panel represent averaged summation (area under the curve) magnitude of activation ± SE for pre-transition (blue) and post-transition (orange); asterisks denote a significant post- vs. pre-transition change (two-tailed, paired *t*-test, *p < 0.0125) that appears below them (in %) in parentheses. Upper row displays pre- and post-transition leveled walking for each of the four muscles, and as expected, pre vs. post are comparable for each of the muscles. Note that in both uphill (middle row) and downhill (bottom row) transitions, the pre-transition (blue curve) is comparable to the leveled condition (blue in the upper row). Post-transition significant changes are evident for inclined transitions (uphill or downhill) for all four muscles apart from the HLR downhill transition (bottom right). TAR, tibialis anterior right; GCR, gastrocnemius right; RFR, rectus femoris right; HLR, hamstring lateralis right. See sections “Results” and “Materials and Methods” for more details.

Consistent with our primary objective and to test our central hypothesis, we examined whether visual cues affect muscle activation patterns (besides modulating behavioral gait measures). To that end, we analyzed pre- and post-transition EMG muscle activation patterns of four different muscles (TAR, GCR, RFR, and HLR). The reasoning for choosing these specific muscles was based on their activation patterns, which were found to be modulated by gravitational surface inclination transitions (Lay et al., 2007; Alexander and Schwameder, 2016; Pickle et al., 2016). As can be appreciated from the top row of Figure 4, each of the four muscles presented a unique activation pattern during the leveled walking stage. Also, as expected for the leveled walking (control condition), the pre- (leveled in blue) and post-transition (leveled in orange), traces were comparable. Furthermore, in the treadmill–vision congruent transitions (uphill T_*U*_V_*U*_ in the second row and downhill T_*D*_V_*D*_ in the third row, Figure 4), there were significant changes between the pre-transition (in blue) muscle activation patterns (note that it is comparable to that of T_*L*_ conditions in the upper row) and the post-transition muscle activation patterns (in orange). It is critical to note that the significant changes took place in all four muscles, except HLR in the T_*D*_V_*D*_ condition (see Table 1 for *p*-values).

**TABLE 1:** The grand averaged (*N* = 12) *p*-value for summation magnitude of muscle activation between pre- and post-transition for all experimental conditions (see upper left corner in Figures 4–7).

	**TAR**	**GCR**	**RFR**	**HLR**
T_*L*_V_*L*_	0.868	0.579	0.290	0.669
T_*L*_V_*U*_	**0**.**005**	**0**.**001**	*0.012*	*0.016*
T_*L*_V_*L*_ increase	**0**.**001**	**0**.**000**	**0**.**000**	**0**.**000**
T_*L*_V_*D*_	0.110	**0**.**004**	0.978	*0.203*
T_*L*_V_*L*_ decrease	0.055	*0.038*	0.858	*0.024*
T_*U*_V_*U*_	*0.021*	*0.013*	**0**.**000**	*0.043*
T_*U*_V_*D*_	*0.026*	**0**.**001**	**0**.**000**	*0.023*
T_*D*_V_*D*_	*0.015*	*0.033*	**0**.**000**	0.173
T_*D*_V_*U*_	*0.011*	0.087	**0**.**000**	*0.048*

*Each row represents one walking condition and each column represents a specific muscle. Significant values (*p* < 0.0125, in bold, corrected for multiple comparisons) indicate a significant change in the summation of magnitude of muscle activation following the transition of the treadmill and/or visual scene. In italics, significance uncorrected for multiple comparisons (*p* < 0.05).*

**FIGURE 5:**
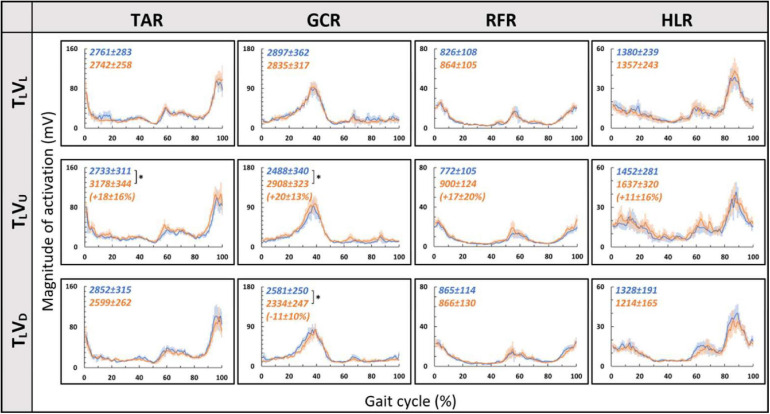
Visual inclinations do not affect muscle activation patterns for leveled treadmill inclination. The *X*-axis represents the percentage of the gait cycle (%), and the *Y*-axis represents the magnitude of muscle activation (mV). Top row—leveled vision (0°, as in Figure 4 top row), middle row—vision up (+10°), and bottom row—vision down (−10°). The blue lines represent the pre-transition and the orange lines represent post-transition activity. Numerical values superimposed on the panels represent the average magnitude of activation ± SE for pre-transition (blue) and post-transition (orange), and in parentheses, the significant changes (in %) between these conditions. *t*-test, *p < 0.0125 (*N* = 12). Despite the significant effects in the magnitude, we found that the EMG temporal patterns were highly comparable; see correlation analyses in Table 2.

We then examined whether parametric changes of the visual cues [i.e., the visual (virtual) inclination] would affect muscle activation patterns for a specific treadmill transition inclination. In contrast to our main hypothesis, we found that virtual visual manipulations of the visual scene did not alter the pattern of muscle activation according to the anticipated gravitational forces, either uphill or downhill. As illustrated in Figure 5, for the leveled treadmill (T_*L*_, at 0°), the parametric manipulation of visual cues did not significantly affect the activation patterns of any of the four muscles as apparent by the similarities between the orange (post-transition) and blue (pre-transition) traces. These qualitatively detected similarities were confirmed by a series of correlation analyses (Table 2). Furthermore, when comparing the post-transition muscle activation pattern between the T_*L*_V_*L*_ condition and T_*L*_V_*U*_ (*r* = 0.90, *p* < 0.01) or T_*L*_V_*D*_ (*r* = 0.93, *p* < 0.01) conditions, significant correlations were seen for all four muscles. As for muscle activation magnitude, during virtual uphill walking, all four muscles exhibited increased activation during the post-transition period compared to the pre-transition period. Addressing the virtual downhill walking, only the GCR exhibited a significant decrease in activation in the post-transition period compared with the pre-transition period.

**TABLE 2:** Averaged normalized correlation coefficients of the muscle activation pattern between pre- and post-transition across all participants (*N* = 12).

	**TAR**	**GCR**	**RFR**	**HLR**
T_*L*_V_*L*_	**1.43 ± 0.1**	**1.42 ± 0.14**	**1.42 ± 0.08**	**1.4 ± 0.09**
T_*L*_V_*U*_	**1.24 ± 0.06**	**1.31 ± 0.12**	**1.39 ± 0.08**	**1.21 ± 0.10**
T_*L*_V_*L*_ increase	**1.2 ± 0.07**	**1.34 ± 0.12**	**1.18 ± 0.1**	**1.09 ± 0.11**
T_*L*_V_*D*_	**1.34 ± 0.11**	**1.22 ± 0.12**	**1.23 ± 0.11**	**1.35 ± 0.07**
T_*L*_V_*L*_ decrease	**0.94** + **0.09**	**1.07 ± 0.13**	**0.95 ± 0.1**	0.830.1
T_*U*_V_*U*_	−0.22 ± 0.11	−0.18 ± 0.1	0.06 ± 0.08	−0.17 ± 0.08
T_*U*_V_*D*_	−0.34 ± 0.06	−0.20 ± 0.07	0.05 ± 0.08	−0.23 ± 0.07
T_*D*_V_*D*_	0.73 ± 0.07	0.39 ± 0.08	0.72 ± 0.1	0.6 ± 0.05
T_*D*_V_*U*_	0.77 ± 0.08	0.41 ± 0.13	0.76 ± 0.11	0.67 ± 0.08

*Average Fisher’s *Z* transformation of Pearson’s correlation coefficients (±SE) for each experimental condition. Values in bold represent a significant correlation (pre- and post-transition) for the temporal patterns of muscle activation, *p*(corr.) = 0.0125. A two-way repeated measures ANOVAs (one for each treadmill inclination) with visual inclination (up, leveled and down) and muscles as factors revealed no significant effects of visual inclination [treadmill leveled: *F*(2,22) = 2.291, *p* = 0.125; treadmill up: *F*(1,11) = 0.735, *p* = 0.410; treadmill down: *F*(1,11) = 3.182, *p* = 0.102]. Regarding the muscle factor, there was an effect of muscle only in the treadmill up [*F*(3,33) = 5.626, *p* = 0.003] and down [*F*(3,33) = 3.327, *p* = 0.031] ANOVAs, but not in the treadmill leveled ANOVA [*F*(3,33) = 0.052, *p* = 0.984]. Note that for each muscle, the Fisher’s *Z* transformation score remains roughly similar for each treadmill inclination regardless of the visual scene inclination. Furthermore, a consecutive two-way repeated measures ANOVA with treadmill inclination (up, leveled, and down) and muscles as factors revealed a significant effect of treadmill inclination [*F*(2,22) = 143, *p* < 0.001]. These findings suggest that the temporal patterns of muscle activation are predominantly defined by body-based cues with possibly only minor reliance on visual cues.*

**FIGURE 6:**
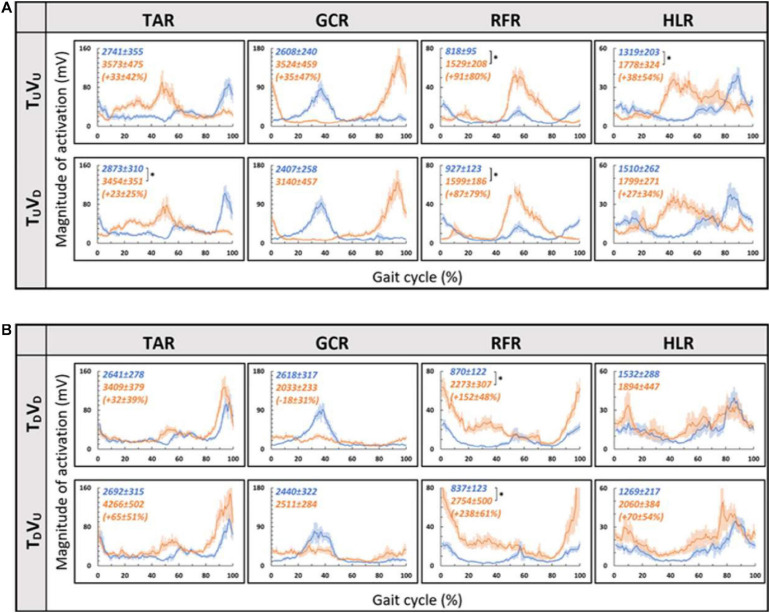
Visual inclinations do not affect muscle activation patterns for uphill **(A)** or for downhill **(B)** treadmill inclination. Conventions as in Figure 5, *p < 0.0125. This demonstrates that patterns of muscle activation during physical uphill walking did not change regardless of conflicting visual cues [panel **(A)**: top row—uphill vision (+10°), bottom row—downhill vision (−10°); compare orange traces in the first and second rows]. Likewise, patterns of muscle activation during physical downhill walking were not affected by conflicting visual cues [panel **(B)**: top row—downhill vision (−10°), bottom row—uphill vision (+10°); compare orange traces in the first and second rows].

As for the treadmill leveled conditions, for both uphill (T_*U*_, at 10°) and downhill (T_*D*_, at −10°) treadmill transitions, the parametric manipulation of visual cues did not affect the activation patterns of any of the four muscles (Figures 6A,B, respectively). In both treadmill transitions, visual inclination did not make a difference (Table 2). In summary, activation patterns that were modulated by gravitational transitions were insignificantly affected by virtual visual cues.

**FIGURE 7:**
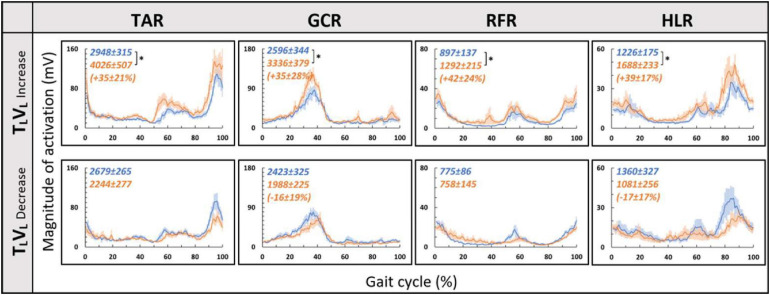
Gait speed does not affect muscle activation patterns for leveled treadmill inclination. Conventions as in Figure 4, *p < 0.0125. Blue lines represent the activity pre-change, and the orange lines represent post-change (increase by ∼15% or decrease by ∼20% from SSV).

Since in the virtual inclination transitions we observed changes in muscle activation magnitude but not in the patterns of activity (Figure 5), we hypothesized that the source of these magnitude changes was driven by the behavioral gait speed changes seen in the incongruent conditions (Figure 2). Therefore, we evaluated muscle activation magnitude in response to mere changes in gait speed when gravitational cues did not change (see section “Materials and Methods” for T_*L*_V_*L*_ increase and T_*L*_V_*L*_ decrease). Indeed, similar effects on muscle activation magnitudes were seen in the incongruent condition in response to changes in gait speed. For example, in condition T_*L*_V_*D*_, the averaged walking speed is decreased (Figure 2) as well as the GCR activation (Figure 5). A similar pattern is illustrated in condition T_*L*_V_*L*_ decrease where the speed is decreased in correlation with GCR activation (Figure 7). By combining the information from Figures 4–7 and Table 2, the observed changes in muscle activation magnitude seen during the incongruent conditions (Figure 5) can be explained by the gait speed changes (Figure 7). This implies that major changes in temporal muscle activation patterns related to uphill/downhill walking, as compared with leveled walking, are likely to rely predominantly on body-based cues.

## Discussion

In this study, we investigated the contribution of vision to locomotion modulation under physical changes. To that end, we manipulated vision independently of body-based cues and measured walking speed and muscle activation. Initially, we replicated recent results that demonstrated that the transition to virtual inclinations modulates locomotion for approximately 20 s (Cano Porras et al., 2020), which was evident by speeding up or slowing down (aka the exertion and braking effects, respectively). Furthermore, in an individual differences analysis, we found that the magnitude of this effect was associated with individual visual field dependency. Lastly, we observed that when transitioning from leveled walking to inclined walking, changes in the temporal pattern of muscle activation were primarily affected by body-based cues and not significantly by vision.

According to the hypothetical internal model of gravity, locomotion modulations regarding gravitational conditions (e.g., walking on inclined surfaces) are based on multiple sensory inputs (visual, vestibular, and proprioceptive). However, the proportional contribution of vision under these conditions is unclear. In order to differentiate between the weights of each sensory channel, there is a need to isolate each of them and examine the behavioral and physiological responses. Thus, in real-world conditions, it is difficult to isolate different sensory modalities. One methodology is to investigate this in specific cohorts such as blind people (Dizio and Lackner, 2000), patients with proprioception deficits (Mullie and Duclos, 2014), or those with vestibular impairments (Potter and Silverman, 1984). Another methodology is to artificially manipulate each sensory cue independently, causing a sensory discrepancy. Regarding visual manipulations, most studies change the speed of the projected visual scene in a virtual reality environment, which ultimately affects the participants’ walking speed. These studies show that increasing the optic flow speed in a VR environment causes participants to decrease their walking speed and vice versa (Lamontagne et al., 2007; O’Connor and Donelan, 2012; de Keersmaecker et al., 2019). A recent study examined vision’s contribution to locomotion employing a different visual paradigm: manipulating physical–visual scene treadmill inclination congruency (Cano Porras et al., 2020). While walking speed was consistently sensitive to visual inputs (supported by our findings as well), it was unclear whether vision’s contribution extends to affect the magnitude or patterns of muscle activation. Our study found that the magnitude of muscle activation was modulated in relation to the walking speed changes following visually induced simulated virtual inclinations. Such adjustments are in accordance with *indirect prediction* mechanisms, i.e., relying on prior experience to activate preprogrammed gait control patterns that are consistent with expected gravity-based changes (adjusting walking speed according to walking at virtual inclinations), similar to changes that occur following visual flow speed manipulations (Snaterse et al., 2011; O’Connor and Donelan, 2012; Cano Porras et al., 2020). However, in contrast to changes in gait speed, we found that the pattern of muscle activation during the gait cycle was dominated by body-based cues responding to the actual gravity-related (physical) forces but less dependent on visual cues. This is demonstrated by the observed shift in peak activation during a real gravitational change (Figure 4) but not when the change is only visual (Figure 5). These results are quantitatively corroborated by a high correlation between activation patterns of post- and pre-transition periods (Table 2). Thus, our findings suggest that vision’s contribution is predominantly involved in dynamic adjustments during indirect prediction for approximately 20 s after a perturbation.

While we found that visual cues affect locomotion for a brief period after perturbations occur (i.e., virtual inclination transitions), the roles of proprioception and vestibular cues in controlling locomotion are continuous and likely to be associated with gait speed. Overall, locomotion relies on feedback (e.g., from joint mechanoreceptors that detect gravitational changes) and feedforward (e.g., anticipating center-of-mass change from prior experience) (Riemann and Lephart, 2002; Seidler et al., 2004; Maeda et al., 2019) mechanisms. For example, visual inputs are used in the process of planning locomotion to create a model of the environment in which the walking would occur, while proprioception is essential during the execution of the movement to update the feedforward commands derived from the visual inputs (Bard et al., 1995; Sainburg et al., 1995). Regarding body-based cues, it seems that during walking, vestibular signals are downregulated, whereas proprioception dominates the pattern of muscle activation. Overall, neural networks controlling posture and gait are highly dependent on vestibular inputs during slow gait and shift their dependency toward proprioception with increased gait speed (Brandt et al., 1999; Pfaff, 2013; Fabre-Adinolfi et al., 2018). The role of proprioception in these regulations is assumed to be in response to external and internal stimuli (Janshen et al., 2017). While we found that gait speed was affected by virtual inclination transitions, probably due to expectations for gravitational changes (i.e., feedforward effects), proprioception governed the muscle activation patterns, which were not affected following virtual visual inclination manipulations. We demonstrated that before adjusting to a complex mechanism such as walking, the pattern of muscle activation only changes after physical mechanoreceptor-related cue has occurred. We speculate that visual inputs have minimal impact, if any, on neuronal activations that define muscle synergies (Lay et al., 2007; Pickle et al., 2016) and temporal muscle activation pattern (Stokes et al., 2017; Cano Porras et al., 2019) characteristic of inclined locomotion. Interestingly, other studies had demonstrated the involvement of visual flow in altering the magnitude of muscle activation during perturbed walking (i.e., when balance control tasks were involved) (O’Connor and Donelan, 2012). For example, Cano Porras et al. recently reported that mere downward visual perturbations (platform “drops” down) generated in a VR system can trigger characteristic muscle activation patterns in standing individuals (Cano Porras et al., 2021). In summary, while visual cues have a transiently significant contribution that wears off (Schindlbeck et al., 2018), body-based cues continuously adjust during walking in response to walking speed.

It is unclear to what extent visual field dependency is related to or affects locomotion. Patient populations with deficiencies in the central nervous system and locomotion deficits show high visual field dependency (measured by subjective visual vertical), probably compensating for their deficits [e.g., Parkinson’s disease (Bonan et al., 2006, 2007), post-stroke (Crevits et al., 2007), multiple sclerosis (Slaboda et al., 2013), and cerebral palsy (Kim et al., 2015)]. Assessments of visual dependency include tests based on visual–vestibular conflicts such as measuring visually induced illusory perception of self-motion, known as vection (Lê and Kapoula, 2008), and the Romberg’s test that measures visually assisted postural stability and aims to identify the influence of vision on postural control by comparing how much the body sways with eyes opened vs. closed (Rothacher et al., 2018). In addition to the rod and frame test used in our study, Rothacher et al., used these two tests and compared them with locomotive outcomes. Out of the three tests, they found that locomotive outcome showed the highest correlation to the rod and frame test by demonstrating that high visual field-dependent participants (as revealed in the rod and frame test) were the most sensitive to visual manipulations in a VR environment. In our study, in line with earlier findings, we found that visual field dependency [as measured by the rod and frame lab-based psychophysical test; Lopez et al. (2006), Isableu et al. (2008), and Bagust (2013)] was significantly correlated with locomotive measures. Specifically, individuals with higher visual field dependency showed higher percentage of change in gait speed to virtually induced inclinations (e.g., treadmill up vision down). Together, these findings support the hypothesis that visual field dependency and locomotion outcomes such as visually dependent gait speed or trajectories may rely on associated mechanisms. There are several possible limitations to our study. First, our study included a relatively small sample size which may limit the generalization of our results. Second, there are limitations that are related to the use of VR platform and how they may reflect real-world situations. In this study, we aimed to evaluate the contribution of visual cues in regard to the body-based cues while walking on inclined surfaces. We used a self-paced treadmill with a fully immersive projected visual scene, which was shown to have high ecological validity (Plotnik et al., 2015). At the same time, transitions to inclined walking were presented within time windows of 5 s, while in real life, the walker can anticipate the “approach” of the inclination ahead of time, based on visual cues from the environment. Despite this limitation, the results of the present paradigm clearly discern between the roles of vision and body-based cues in adjusting gait behavior with reference to inclinations. Finally, all our participants were exposed to this large-scale VR system for the first time during this experiment which may have generated excitement or other psychological effects. To mitigate this, we took measures to confirm with the participants that they felt well, and we started the experiments with several acclimation trials.

In this study, we examined the effect of visual cues while walking on inclined surfaces in a young, healthy population, to explore how muscle activation is affected by visual cues. We found that the magnitude of the behavioral effect (i.e., change in gait speed) was associated with individual visual field dependency. Furthermore, we observed that when transitioning from leveled to inclined walking, the pattern of muscle activation was affected mainly by body-based cues and not by visual cues. It is still unclear whether the anticipatory change in gait speed relies on an estimate of gravity to determine the appropriate direction and magnitude. A more profound comprehension of locomotion in healthy populations may contribute to understanding pathologies in neurological disorders such as Parkinson’s disease and stroke (commonly presenting with gait disorders). Such knowledge may also be used in combination with VR systems for motor rehabilitation in such conditions (Lamontagne et al., 2007; Darekar et al., 2015; Cano Porras et al., 2018, 2019; de Keersmaecker et al., 2019) or in persons with other walking disabilities.

## Data Availability Statement

The original contributions presented in the study are included in the article/Supplementary Material, further inquiries can be directed to the corresponding author/s.

## Ethics Statement

The studies involving human participants were reviewed and approved by the Sheba Medical Center. The patients/participants provided their written informed consent to participate in this study.

## Author Contributions

MP, SG-D, and AB developed the research question and methodologies. AB and SZ recruited the participants and ran the experiments. OB-G and DCP programmed the calculation tools. AB and AW analyzed the data. AB was the main author, MP, and SG-D made substantial revisions, and all co-authors have read and approved the final manuscript.

## Conflict of Interest

The authors declare that the research was conducted in the absence of any commercial or financial relationships that could be construed as a potential conflict of interest.

## Supplementary Material

Supplementary Figure 1 Deriving gait speed-related variables. Depicts an example of responses to virtual uphill [T_*L*_V_*U*_; panel **(A)**] and downhill [T_*L*_V_*D*_; panel **(B)**] transitions from one participant (OM018). The vertical orange line represents the transition, and the horizontal orange line represents steady-state velocity (SSV). On these traces, the peak/trough were identified (green arrows), and the time from transition was calculated (gray line). Units used for magnitude were the relative change (%) in comparison to the SSV values. The second parameter is the time of maximal change.

Supplementary Figure 2 A graphical representation of the right gastrocnemius (GCR) muscle from 24 gait cycles obtained from one participant during the T_*U*_V_*U*_ condition. The *X*-axis shows the sample rate (Hz), and the *Y*-axis shows muscle activation (mV). As can be seen, the pattern of activation remains similar for all the gait cycles pre-transition (purple frame) and post-transition (orange frame); the green frame represents the 5 s transition.

Supplementary Figure 3 Pre- vs. post- EMG right Gastrocnemius activation patterns for visual-physical congruent uphill walking. Grand average (across participants, *N* = 12) of muscle activation patterns (*Y*-axis, in mV) plotted against a time-normalized gait cycle on the *X*-axis (in % of the gait cycle) in blue (shaded areas represent SE) for pre-transition and in orange (shaded areas represent SE) for 20 s post-transition. Numerical values at top left represent averaged summation (area under the curve) magnitude of activation ± SE for pre-transition (blue) and post-transition (orange), asterisks denote a significant post- vs. pre-transition change (two-tailed, paired *T*-test, ^∗^*P* < 0.05). The normalized change appears below (in %) in parentheses.

Supplementary Table 1 No significant difference in the patterns of muscle activation between Cano Porras 2020 and the recent study. Each row represents a walking condition (see section “Materials and Methods” for details). The 2nd and 3rd columns represent the average values for the relative peak change from SSV (%) ± SE. The 5th and 6th columns represent the average time of peak effect (seconds) for each study ± SE. The 4th and last columns represent the *p*-value obtained from a paired two-tailed *t*-test. ^∗^ (*N* = 14), ^∗∗^ (*N* = 12). A Kruskal–Wallis test showed that the magnitude of relative speed change was significantly affected by condition (*p* = 0.002). *Post hoc* analyses revealed that the effect originated from the difference in relative speed change between TUVD and (i) TLVD (*p* = 0.04), and (ii) TDVU (*p* = 0.03). All other dichotomic comparisons were not found statistically significant (*p* ≥ 0.185).

Supplementary Video 1 The video insert depicts the behavior effects of visually induced downhill walking. Initially, the person is walking with the treadmill, and the visual scene leveled. Once the person reaches steady-state velocity and maintains it for 12 s, a downward transition of the visual scene occurs, while the treadmill remains leveled. Note how she decreases her gait speed to counteract the expected gravitational forces (i.e., the braking effect). Eventually, body-based cues govern, and gait speed returns to the previous steady-state values.
