# Supplementary material for: Vision Affects Gait Speed but not Patterns of Muscle Activation During Inclined Walking—A Virtual Reality Study
Source: Front Bioeng Biotechnol. 2021 Apr 9;9:632594. doi: 10.3389/fbioe.2021.632594 (PMC8062981; doi:10.3389/fbioe.2021.632594)
Supplement: Supplementary Figure 1 — Deriving gait speed-related variables. Depicts an example of responses to virtual uphill [TLVU; panel (A)] and downhill [TLVD; panel (B)] transitions from one participant (OM018). The vertical orange line represents the transition, and the horizontal orange line represents steady-state velocity (SSV). On these traces, the peak/trough were identified (green arrows), and the time from transition was calculated (gray line). Units used for magnitude were the relative change (%) in comparison to the SSV values. The second parameter is the time of maximal change. [file Data_Sheet_1.docx]

**Supporting material for the *Methods* section**


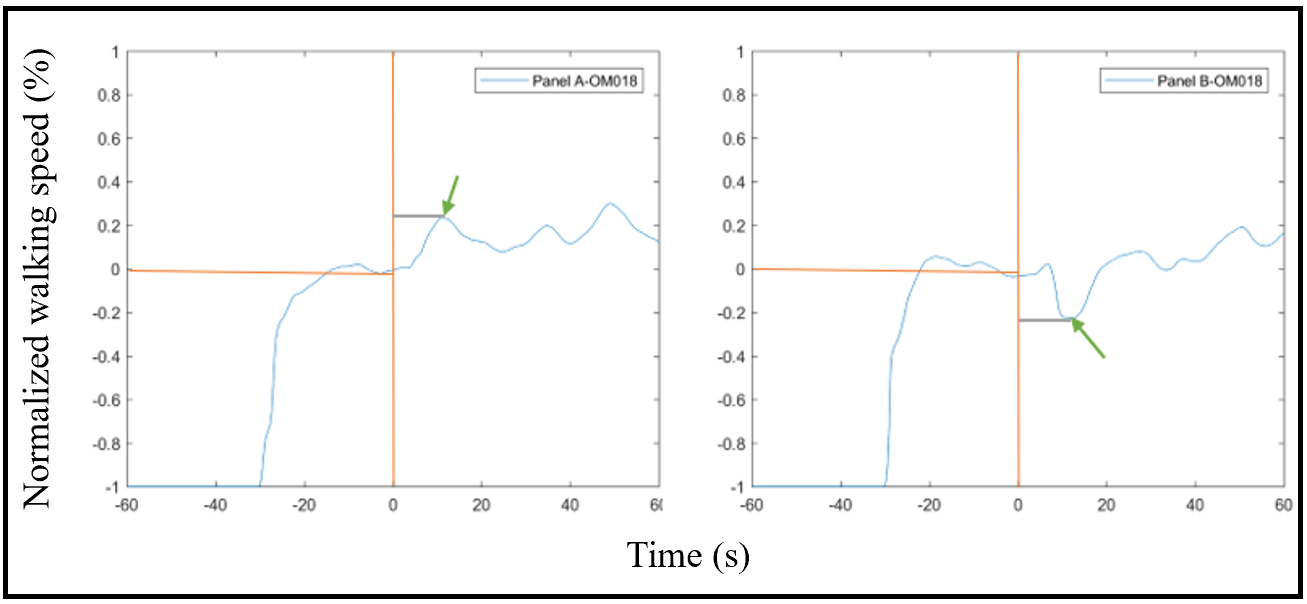


**Figure S-1.** Deriving gait speed-related variables. Fig. S-1 depicts an example of responses to virtual uphill (T_L_V_U_; panel A) and downhill (T_L_V_D_; panel B) transitions from one participant (OM018). The vertical orange line represents the transition, and the horizontal orange line represents steady-state velocity (SSV). On these traces, the peak/trough were identified (green arrows), and the time from transition was calculated (grey line). Units used for magnitude were the relative change (%) in comparison to the SSV values. The second parameter is the time of maximal change (c.f., Fig. S-1).

*Electromyography (EMG) analysis:*

Figure S-2 shows an example from one participant of gait cycles obtained10 seconds before the transition (when the participant walked in SSV), during the 5 seconds of transition and 18 seconds post-transition According to this example of T_U_V_U_ condition, the EMG pattern through all the frames is roughly similar before the transition, compared to a gradual change of EMG pattern during the transition. It is critical to note that after the transition, the EMG pattern is constant throughout the selected gait cycles.

From the time-scaled EMG traces, we grand averaged all the pre-data and post-data separately (see Fig. S-3). With the averaged EMG traces, we computed the following two parameters:

**(1) Magnitude** – the summation (i.e., area under the curve) of the EMG traces. In this example, the MAG values are depicted in the upper left side of the panel (Fig S-3). Also, the normalized percentage of the difference is stated in parenthesis, where a significant change was observed between post- and pre- transitions.

*Normalization for summation of the magnitude of activation*

Due to various individual differences (e.g., sweating, hair, conductivity), the obtained results might not be evenly scaled. . To address this problem, each participants’ results were normalized per condition according to the value of the magnitude of activity in the pre-transition condition.

**(2) Similarity of EMG activation pattern** – The post transition EMG trace was correlated with the pre-transition EMG trace (Pearson Correlation) for each participant for each condition. A high correlation value indicates a small change in the pattern of activation. In this example (Fig S-3), the correlation value is -0.17±0.08 between the orange and blue trace. This low value indicates a significant change in EMG activation pattern seen between leveled walking and uphill walking in the gastrocnemius (GCR) muscle.


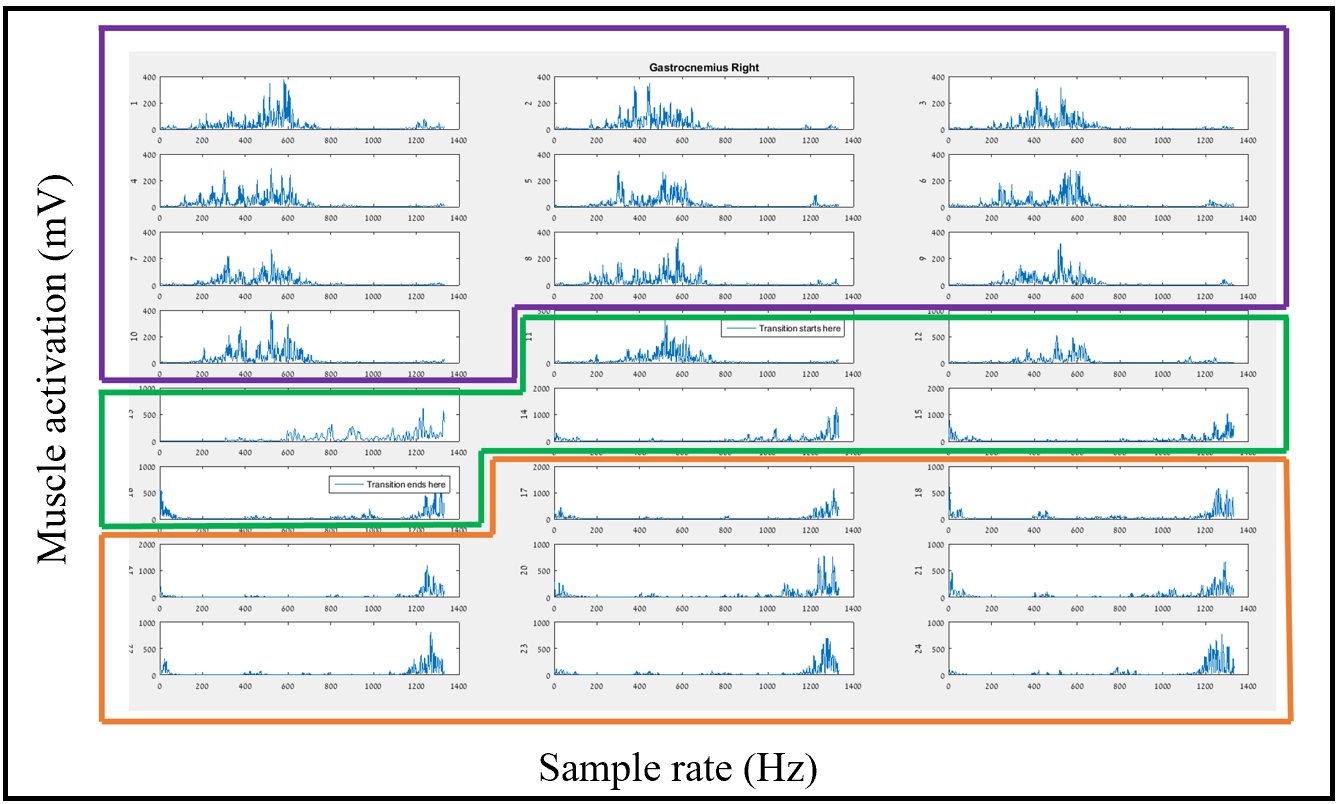


**Figure S-2**. A graphical representation of the right gastrocnemius (GCR) muscle from 24 gait cycles obtained from one participant during the T_U_V_U_ condition. The X-axis shows the sample rate [Hz], and the Y-axis shows muscle activation [mV]. As can be seen, the pattern of activation remains similar for all the gait cycles pre-transition (purple frame) and post-transition (orange frame); the green frame represents the 5s transition.


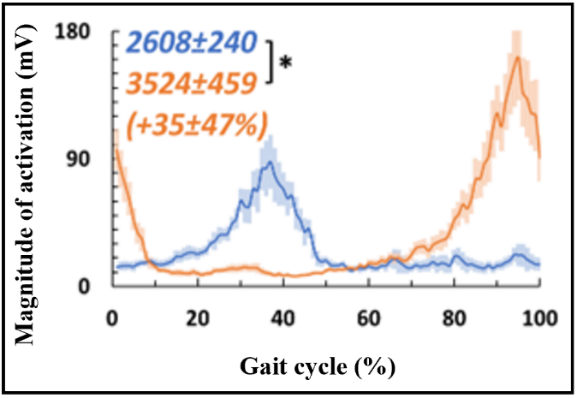


**Figure S-3.** Pre- vs. post- EMG right Gastrocnemius activation patterns for visual-physical congruent uphill walking. Grand average (across participants, N=12) of muscle activation patterns (Y-axis, in mV) plotted against a time-normalized gait cycle on the X-axis (in % of the gait cycle) in blue (shaded areas represent SE) for pre-transition and in orange (shaded areas represent SE) for 20 seconds post-transition. Numerical values at top left represent averaged summation (area under the curve) magnitude of activation ± SE for pre-transition (blue) and post-transition (orange), asterisks denote a significant post- vs. pre-transition change (two-tailed, paired T-test, P<0.05=*). The normalized change appears below (in %) in parentheses.

**Supporting material for the *Results* section**

*Detailed description of the results presented in Figure 2*

For the T_U_ conditions (when treadmill transition changed to 10° uphill, upper row of Figure 2), there was a gradual decrease in gait speed during the first 20 seconds after transition (cf. left T_U_V_U_ panel with smallest decrease, middle T_U_V_L_ panel with a medium decrease, and right T_U_V_D_ panel with largest decrease after which the gait speed returned roughly to physical-based gait speed regardless of the visual (virtual) inclination. Similarly, for the T_L_ conditions (when the treadmill remained leveled at 0°, middle row of Figure 2), there was a gradual change in gait speed in the first 20 seconds after the virtual transition (cf. left T_L_V_U_ panel with an increase (visually induced exertion effect), middle T_L_V_L_ panel with no change, and right T_L_V_D_ panel with a decrease (visually induced braking effect)) after which the gait speed returned roughly to gravitational-based gait speed regardless of the visual (virtual) inclination. For the T_D_ conditions (when treadmill transition changed to -10° downhill, lower row of Figure 2), the same pattern of a gradual change in gait speed in the first 20 seconds after the virtual transition was observed (cf. left T_D_V_U_ panel with an increase, middle T_D_V_L_ panel with a smaller increase and right T_D_V_D_ panel with no change). Following that, gait speed returned roughly to the gravitational-based level regardless of visual inclination.

*Comparison with Cano-Porras et al* (Cano Porras et al., 2020)- Table S-1provides comparison between the results of the present study and the results of an earlier study conducted in this laboratory using the same paradigm. The following data confirms the reproducibility of results using the present virtual reality paradigm.

|  | Magnitude of relative speed change (%) | | | Time of maximal effect (s) | | |
| --- | --- | --- | --- | --- | --- | --- |
|  | Cano Porras 2020* | Present study** | P-value | Cano Porras 2020* | Present study** | P-value |
| T_U_V_U_ | 32.8±15.4 | 25.4±12 | 0.18 | 14.4±6.8 | 13.5±5.1 | 0.69 |
| T_D_V_D_ | 13.9±23.3 | 18.8±14.1 | 0.47 | 17.2±8.2 | 22.2±8.3 | 0.15 |
| T_L_V_U_ | 15.9±11.2 | 17.9±8.9 | 0.64 | 12.3±4.5 | 10.3±2.2 | 0.19 |
| T_L_V_D_ | 20.7±16.8 | 25±15.5 | 0.5 | 7.6±1.9 | 8±2 | 0.59 |
| T_D_V_U_ | 21.6±17.2 | 28.1±20 | 0.38 | 11.6±5.5 | 9.2±2.7 | 0.16 |
| T_U_V_D_ | 62.8±24.7 | 58.3±30.4 | 0.68 | 7.9±1.7 | 7.2±2.3 | 0.38 |

**Table S-1**. No significant difference in the patterns of muscle activation between Cano Porras 2020 and the recent study. Each row represents a walking condition (see methods for details). The 2^nd^ and 3^rd^ columns represent the average values for the relative peak change from SSV (%)±SE. The 5^th^ and 6^th^ columns represent the average time of peak effect (seconds) for each study±SE. The 4^th^ and last columns represent the p-value obtained from a paired two-tailed t-test. * (N=14), ** (N=12). A Kruskal-Wallis test showed that the magnitude of relative speed change was significantly affected by condition (p=0.002). Post-hoc analyses revealed that the effect originated from the difference in relative speed change between TUVD and (i) TLVD (p=0.04), and (ii) TDVU (p=0.03). All other dichotomic comparisons were not found statistically significant (p≥0.185).

**Supplementary Video 1**:

<https://drive.google.com/file/d/1FLQsbuMKCX7xKWtewG89PfDTI7WSM19I/view?usp=sharing>

The video insert depicts the behavior effects of visually induced downhill walking. Initially, the person is walking with the treadmill, and the visual scene leveled. Once the person reaches steady-state velocity and maintains it for 12 seconds, a downward transition of the visual scene occurs, while the treadmill remains leveled. Note how she decreases her gait speed to counteract the expected gravitational forces (i.e., the braking effect). Eventually, body-based cues govern, and gait speed returns to the previous steady-state values.
